# The Late Sir Philip Crampton, M.D.

**Published:** 1858-09

**Authors:** 


### NECROLOGICAL NOTICES.

The Late Sir Philip Crampton, M.D., Bart, F.R.S. — The *Medical Times and Gazette* brings us the news of the death of this eminent man, on the 10th of June, 1858, at the advanced age of eighty-one. He entered the army when very young, and at twenty-one was appointed Surgeon to the Meath Hospital, a position which he held for nearly sixty years. Two years later he took the degree of M.D. at the University of Glasgow, and soon published "An Essay on Entropion, or Inversion of the Eyelids," being at the time Assistant Surgeon to the Westmoreland Lock Hospital. Mr. Crampton was the first, in connection with Peter Harkan, to form a private school of Anatomy and Surgery in Dublin; the former taking the department of Surgery. His practice, from this time, rapidly increased, and he soon gave up private instruction. He was the discoverer of the muscle in the eye of birds, by which changes in its refractive power is produced, and to which Kölliker has given the name of "Musculus Cramptonianus." For this, Mr. Crampton was created a Fellow of the Royal Society, and about the same time was appointed Surgeon-General to the Forces. In the reign of George IV. he received the appointment of Surgeonin-Ordinary to the King of Ireland, and in 1839 he was honored by the present Regent with the dignity of a Baronet of the United Kingdom. He was president of several, and a member of many learned societies. Medical periodical literature owes much to his pen, and his contributions may be found, principally, in the *Medico-Chirurgical Transactions*, the *Dublin Hospital Reports*, and the *Dublin Journal of Medical Science*. Mr. Crampton was devoted to his profession, in which he held the first rank; and his genius, talents, and scientific attainments were only equaled by his kind and affectionate disposition, which made him universally beloved.

